# Bypassing reproductive barriers in hybrid seeds using chemically induced epimutagenesis

**DOI:** 10.1093/plcell/koab284

**Published:** 2021-11-18

**Authors:** Jonathan Huc, Katarzyna Dziasek, Kannan Pachamuthu, Tristan Woh, Claudia Köhler, Filipe Borges

**Affiliations:** 1 Institut Jean-Pierre Bourgin, INRAE, AgroParisTech, Université Paris-Saclay, 78000, Versailles, France; 2 Department of Plant Biology, Uppsala Biocenter, Swedish University of Agricultural Sciences, Linnean Center of Plant Biology, Uppsala, Sweden; 3 Max Planck Institute of Molecular Plant Physiology, Potsdam-Golm, Germany

## Abstract

The triploid block, which prevents interploidy hybridizations in flowering plants, is characterized by a failure in endosperm development, arrest in embryogenesis, and seed collapse. Many genetic components of triploid seed lethality have been successfully identified in the model plant *Arabidopsis thaliana*, most notably the paternally expressed genes (PEGs), which are upregulated in tetraploid endosperm with paternal excess. Previous studies have shown that the paternal epigenome is a key determinant of the triploid block response, as the loss of DNA methylation in diploid pollen suppresses the triploid block almost completely. Here, we demonstrate that triploid seed collapse is bypassed in Arabidopsis plants treated with the DNA methyltransferase inhibitor 5-Azacytidine during seed germination and early growth. We identified strong suppressor lines showing stable transgenerational inheritance of hypomethylation in the CG context, as well as normalized expression of PEGs in triploid seeds. Importantly, differentially methylated loci segregate in the progeny of “epimutagenized” plants, which may allow epialleles involved in the triploid block response to be identified in future studies. Finally, we demonstrate that chemically induced epimutagenesis facilitates hybridization between different Capsella species, thus potentially emerging as a strategy for producing triploids and interspecific hybrids with high agronomic interest.

## Teaser

Genome-wide loss of DNA methylation induced by 5-Azacytidine allows interploidy and interspecific hybridization barriers to be bypassed in Arabidopsis and Capsella.

##  

**Figure:**
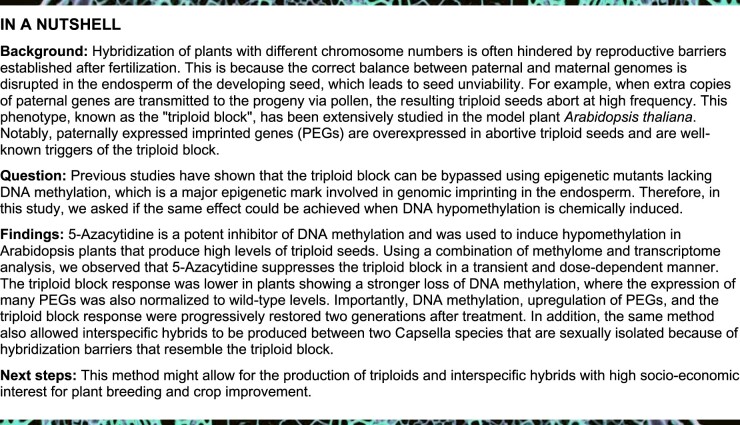


## Introduction

Early studies in plants provided some of the first evidence that distinctive phenotypes are dependent on the nature and dosage of parental chromosomes (Blakeslee et al., 1920; Belling and Blakeslee, 1923). This phenomenon, currently known as heterosis, is often observed in interploid and interspecific hybrids that display phenotypic values exceeding those in their parents, but its genetic and epigenetic basis remain poorly understood (Birchler et al., 2010; Hochholdinger and Baldauf, 2018). Plant breeders have been exploiting heterosis for thousands of years to create elite varieties of domesticated crops with enhanced growth and yield. However, additional progress has been hindered by the existence of strong reproductive barriers, which prevent heterosis and the rapid introgression of valuable alleles from wild species into domesticated cultivars (Kaneko and Bang, 2014).

In many angiosperms, interploidy crosses between diploid females and tetraploid males lead to abnormal endosperm development and seed collapse, which is known as the “triploid block” response (Köhler et al., 2021). The endosperm of most flowering plants, an essential triploid tissue that nourishes early embryo development in the seed, is formed in the embryo sac by fertilization of the diploid central cell by a haploid sperm cell (Dresselhaus et al., 2016). Endosperm cellularization is a critical event for seed development that relies on the correct balance between the dosage of maternal and paternal genomes (2m:1p) and distinct epigenetic features that are pre-established in the gametes to control genomic imprinting after fertilization (Kawashima and Berger, 2014; Gehring and Satyaki, 2017). Maternally expressed genes encode components of the Polycomb Repressive Complex 2 (PRC2), which is essential to silence the maternal allele of paternally expressed genes (PEGs) via deposition of tri-methylation at lysine 27 of histone 3 (H3K27me3; Köhler and Lafon-Placette, 2015; Gehring and Satyaki, 2017). For a long time, this mechanism was sufficient to explain how imprinted gene expression is established in the endosperm. However, additional models emerged with the discovery of a paternally inherited small RNA-directed DNA methylation pathway that is also involved in genomic imprinting (Calarco et al., 2012; Vu et al., 2013).

The triploid block traces back to the Endosperm Balance Number (EBN) hypothesis, which was developed in the early 1980s in potato (*Solanum tuberosum*) and then extended to many other crops (Johnston and Hanneman, 1982; Ehlenfeldt and Hanneman, 1984). The EBN, or “effective ploidy”, is the ratio between maternal and paternal chromosomes or genetic factors required for development of a normal seed (Johnston and Hanneman, 1982; Ehlenfeldt and Hanneman, 1984; Carputo et al., 1999, 1997). These studies are highly relevant for plant breeding, as the success of interspecific and intergeneric hybridizations may be predicted and manipulated based on the EBN of each parent (Tonosaki et al., 2018), although this system seems to be restricted to only certain genera (Carputo et al., 1999). Additional breeding strategies to overcome hybridization barriers require ovule/ovary cultures and embryo rescue techniques prior to seed collapse, which have limited efficiency depending on the species (Eeckhaut et al., 2006; Sauer and Friml, 2008; Cisneros and Tel-Zur, 2010). In the model plant *Arabidopsis thaliana*, loss-of-function mutations in PEGs are able to suppress the triploid block (Kradolfer et al., 2013; Wolff et al., 2015; Batista et al., 2019). This clearly indicates that endosperm failure during interploidy hybridization results from upregulation of PEGs, which is also observed in crosses between different Arabidopsis species (Josefsson et al., 2006; Kirkbride et al., 2015) and may play role in establishing interspecific hybridization barriers as well (Lafon-Placette et al., 2018). However, its potential application in plant breeding is limited, as imprinted genes are generally not well conserved (Kradolfer et al., 2013; Rodrigues and Zilberman, 2015), often preventing findings from Arabidopsis to be directly tested in crops. More recently, several studies have shown that the paternal epigenome triggers the triploid block response in Arabidopsis, which provided new ideas for plant breeding. The loss of DNA and histone methylation, as well as small-interfering RNAs in diploid pollen restored the viability of triploid seeds almost completely (Schatlowski et al., 2014; Erdmann et al., 2017; Jiang et al., 2017; Borges et al., 2018; Martinez et al., 2018; Satyaki and Gehring, 2019), suggesting that the paternal epigenome mediates genomic imprinting and endosperm balance in the developing seed.

Here, we show that transient genome-wide epimutagenesis induced by 5-Azacytidine, a chemical inhibitor of DNA methylation, allows the triploid block to be bypassed in Arabidopsis. We identified and characterized strong epigenetic suppressors of the triploid block response, showing stable transgenerational loss of CG methylation and downregulation of imprinted genes that are well-known triggers of triploid seed collapse. Finally, we demonstrate that epimutagenesis induced by 5-Azacytidine allows hybridization barriers in crosses between Capsella (shepherd’s purse) species to be bypassed, thus potentially emerging as a method to facilitate the production of triploid plants and interspecific hybrids of high socio-economic interest for agriculture and crop improvement.

## Results

### Exposure to 5-Azacytidine during seed germination and early growth allows the triploid block to be bypassed

Chemical inhibition of DNA methyltransferases has been widely used to study the function of DNA methylation in several plant systems (Pecinka and Liu, 2014). For instance, cytosine analogs such as 5-Azacytidine and Zebularine are incorporated into newly replicated DNA, but do not become methylated (Pecinka and Liu, 2014), causing DNA methylation to be passively erased during cell divisions (Jones and Taylor, 1980; Creusot et al., 1982; Santi et al., 1984). Recent whole-genome analysis of DNA methylation in Arabidopsis has shown that both chemicals lead to the widespread loss of DNA methylation in all sequence contexts, and in a dose-dependent manner (Griffin et al., 2016), which can be stably inherited into subsequent generations or fully restored before fertilization (Akimoto et al., 2007; Baubec et al., 2009).

**Figure 1:**
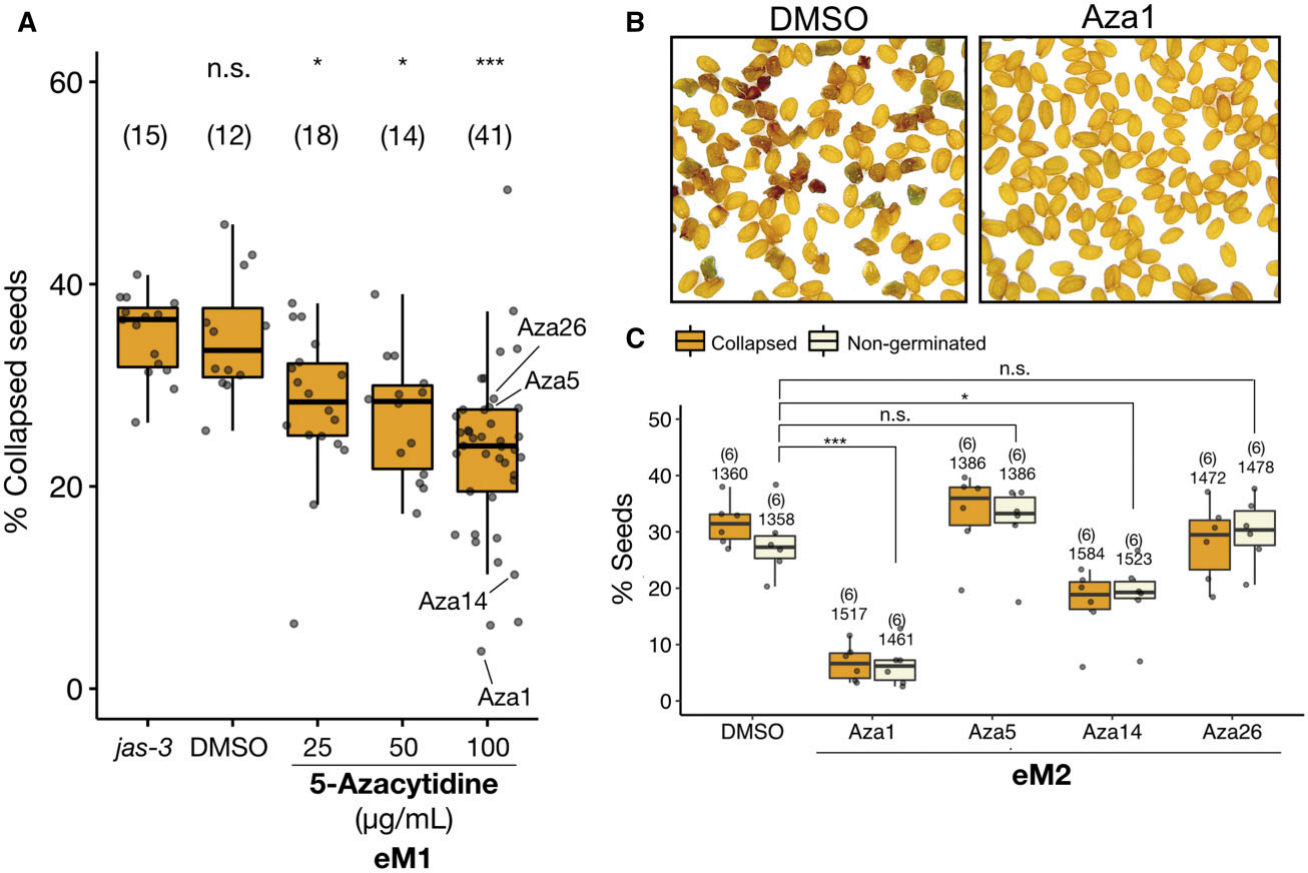
The triploid block is suppressed in *jas-3* plants treated with 5-Azacytidine. A, The triploid block response in *jas-3* mutants was quantified by counting the number of aborted seeds in five siliques of untreated plants (*jas-3*), the DMSO controls (DMSO), and plants exposed to three different concentrations of 5-Azacytidine (25, 50, and 100 µg·mL^−1^) in the eM1 generation (epiMutagenized population, first generation). Numbers above each box represent the number of individual plants used. Statistically significant differences in the percentage of collapsed seeds were calculated by ANOVA with a post hoc Dunnett test, using *jas-3* as the reference group (n.s. is not significant, *P ≤ 0.05, ***P ≤ 0.001). Boxes represent the interquartile range (IQR) showing the lower (Q1) and upper (Q3) quartiles surrounding the median (central line), and whiskers represent the minimum (Q1 −1.5*IQR) and maximum (Q3 + 1.5*IQR) values. B, Representative images of seeds from the DMSO controls and the strong suppressor line Aza1 in the eM2 generation, showing a decrease in the level of seed abortion in the suppressor line. C, Seed abortion (orange) and germination (ivory) assays were performed in the second generation after treatment with 5-Azacytidine (eM2) to evaluate the transgenerational stability of the suppressive effect. Numbers above each box indicate the number of siblings (top) and total number of seeds (bottom) counted. Statistically significant differences in the percentage of nongerminated seeds were calculated by ANOVA with a post hoc Dunnett test, using DMSO as the reference group (n.s. is not significant, *P ≤ 0.05, ***P ≤ 0.001). Boxes represent the interquartile range (IQR) showing the lower (Q1) and upper (Q3) quartiles surrounding the median (central line), and whiskers represent the minimum (Q1 −1.5*IQR) and maximum (Q3 + 1.5*IQR) values.

In order to test if epigenetic variation induced by 5-Azacytidine allows the triploid block response to be suppressed, we used the Arabidopsis mutant *jason* (*jas*; Erilova et al., 2009; Storme and Geelen, 2011). In our growth conditions, plants homozygous for the *jas-3* allele in the Col-0 background produce 30%–40% diploid pollen (Supplemental Figure S1), while the female gametophyte is haploid. Thus, the triploid block response may be quantified after self-fertilization. In untreated *jas-3* plants and dimethyl sulfoxide (DMSO)-treated controls, triploid seed abortion varied between 30% and 40% (Figure  1A), thus reflecting the amount of diploid pollen in these plants (Supplemental Figure S1). Strikingly, plants treated with different concentrations of 5-Azacytidine showed a variable and dose-dependent effect in the triploid block response (Figure  1A), which was significantly reduced to ∼20% in plants treated with 100 µg·mL^–1^ of the chemical (Figure  1A). In particular lines (e.g. Aza1), seed collapse was reduced to <10% (Figure  1A), while the amount of diploid pollen remained unchanged (Supplemental Figure S1), thus suggesting strong suppression of the triploid block response. Close inspection of the seed set showed the presence of enlarged seeds (Figure  1B) that were routinely confirmed to be triploids by flow cytometric analysis of ploidy (Supplemental Figure S2).

We then selected diploid seeds from individual 5-Azacytidine-treated lines to inspect the stability of the suppressive effect in the next generation (Supplemental Figure S3), which will be hereafter designated as eM2 (epimutagenized population, second generation). We compared seeds from six individual eM2 plants of two suppressor lines (Aza1 and Aza14) and two nonsuppressor lines (Aza5 and Aza26) to the DMSO controls, showing that seed abortion rates were similar to those observed in eM1 plants that had been directly exposed to the chemical (Figure  1C). Taken together, these results demonstrate that exposing *jas-3* plants to 5-Azacytidine during seed germination and early growth allows the triploid block response to be bypassed at variable levels, in a dose-dependent and transgenerational manner.

In Arabidopsis, known epigenetic suppressors of the triploid block show an effect only when they are paternally inherited (Schatlowski et al., 2014; Borges et al., 2018; Martinez et al., 2018; Satyaki and Gehring, 2019). We therefore asked if there is also a parental effect in triploid block suppression caused by 5-Azacytidine treatment. To test this hypothesis, we performed reciprocal crosses between DMSO control plants and siblings of the strong suppressor line Aza1 in the eM2 generation. Indeed, we found that the suppressive effect caused by 5-Azacytidine is transmitted via the paternal genome (Supplemental Figure S4).

### Genome-wide CG hypomethylation is observed in the suppressor lines

**Figure 2:**
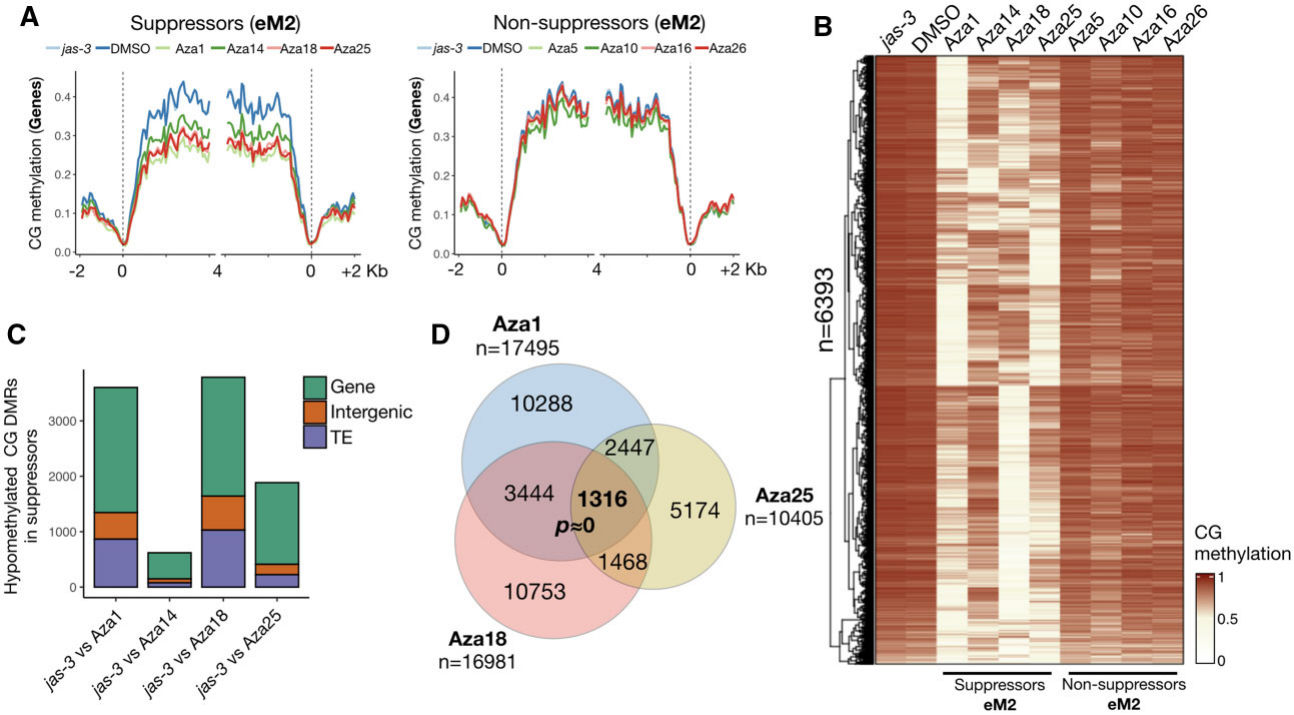
Transgenerational inheritance of CG hypomethylation occurs specifically in the suppressor lines. A, Metaplots show CG methylation levels at protein-coding genes annotated according to the TAIR10 reference genome and aligned at the 5′- and 3′-ends (dashed lines). Average CG methylation was calculated for 100-bp intervals and plotted for untreated *jas-3* and the DMSO controls and for suppressor and nonsuppressor lines in the eM2 generation, showing that the loss of CG methylation occurs specifically in the suppressor lines. B, Heatmap representation of CG methylation levels at hypomethylated CG DMRs detected in the suppressor lines (Aza1, Aza14, Aza18, and Aza25) compared to the untreated control *jas-3*. Average CG methylation mapping to these DMRs is presented as a heatmap for untreated *jas-3* (two replicates) and the DMSO controls (two replicates), suppressors, and nonsuppressors. C, Hypomethylated CG DMRs detected in each suppressor line were mapped to the genomic features annotated in the TAIR10 reference genome, showing that the majority of DMRs overlap with protein-coding genes and transposable elements (TEs). D, Venn diagram shows a significant overlap between differentially methylated 100-bp bins detected in the three strongest suppressor lines (Aza1, Aza18, and Aza25). The statistical significance of the observed overlap was calculated using the R package SuperExactTest (Wang et al., 2015).

To test whether the suppression of the triploid block in 5-Azacytidine-treated *jas-3* plants correlated with a transgenerational loss of DNA methylation, we performed whole-genome bisulfite sequencing (WGBS) (Supplemental Data Set S1). We performed comparative methylome analyses in the eM2 generation of the strong suppressor lines Aza1 (3.7% collapsed seeds), Aza14 (11.3%), Aza18 (6.6%), and Aza25 (6.3%) and the nonsuppressor lines Aza5 (27.8%), Aza10 (30,7%), Aza16 (26.9%), and Aza26 (28.7%), which showed levels of seed collapse closer to the controls *jas-3* (mean 33.7%) and DMSO (mean 33.4%). Indeed, in the suppressor lines, the strong loss of CG methylation was observed at protein-coding genes and transposable elements compared to the untreated *jas-3* and DMSO controls (Figure  2A; Supplemental Figure S5). Analysis of differentially methylated regions (DMRs) led to the identification of 6,393 hypomethylated CG DMRs among all suppressor lines (Figure  2B), which occurred primarily at protein-coding genes (Figure  2C; Supplemental Data Set S2). Interestingly, this analysis also showed variable patterns of hypomethylation in the different suppressors (Figure  2B), as the overlap between the three strongest suppressors (Aza1, Aza18, and Aza25) is limited to ∼1/10 of the amount of DMRs detected in each individual line (Figure  2D). In contrast, CG methylation levels were mostly unchanged in all nonsuppressors (Figure  2A; Supplemental Figure S5), confirming the notion that genome-wide levels of CG methylation correlate with the triploid block response. Importantly, cytosine methylation in the CHG and CHH contexts was mostly unchanged in both suppressor and nonsuppressor lines (Supplemental Figure S5 and Supplemental Data Set S1), and only approximately 100 DMRs were detected in the strongest suppressor lines (Supplemental Figure S6 and Supplemental Data Set S2). This suggests that non-CG methylation was rapidly restored in the eM2 generation and is likely not responsible for the suppressive effect.

In DNA methylation mutants, such as *met1* and *ddm1*, ectopic CHG methylation is often observed in gene bodies (Stroud et al., 2013) and may lead to transcriptional gene silencing. Similarly, ectopic CHG methylation was observed at paternally expressed downregulated genes in the endosperm of viable triploid seeds after pollination with diploid *met1* pollen (Schatlowski et al., 2014). It was hypothesized that ectopic CHG methylation at the paternal *met1* genome leads to reduced expression of PEGs during endosperm development, thus contributing to the viability of seeds with paternal excess (Schatlowski et al., 2014). Although this effect was not previously reported in plants treated with 5-Azacytidine (Griffin et al., 2016), we detected 95 hypermethylated CHG DMRs in the eM2 generation of the strongest suppressor line Aza1 (Supplemental Figure S7). This indicates that newly formed epialleles with ectopic CHG methylation are induced by 5-Azacytidine treatment and are stably inherited to the next generation. However, this effect was not consistently observed among the four suppressor lines tested (Supplemental Figure S7), suggesting that ectopic CHG is also not responsible for the suppressive effect.

### The triploid block response and DNA methylation levels are partially restored two generations after 5-Azacytidine treatment

**Figure 3:**
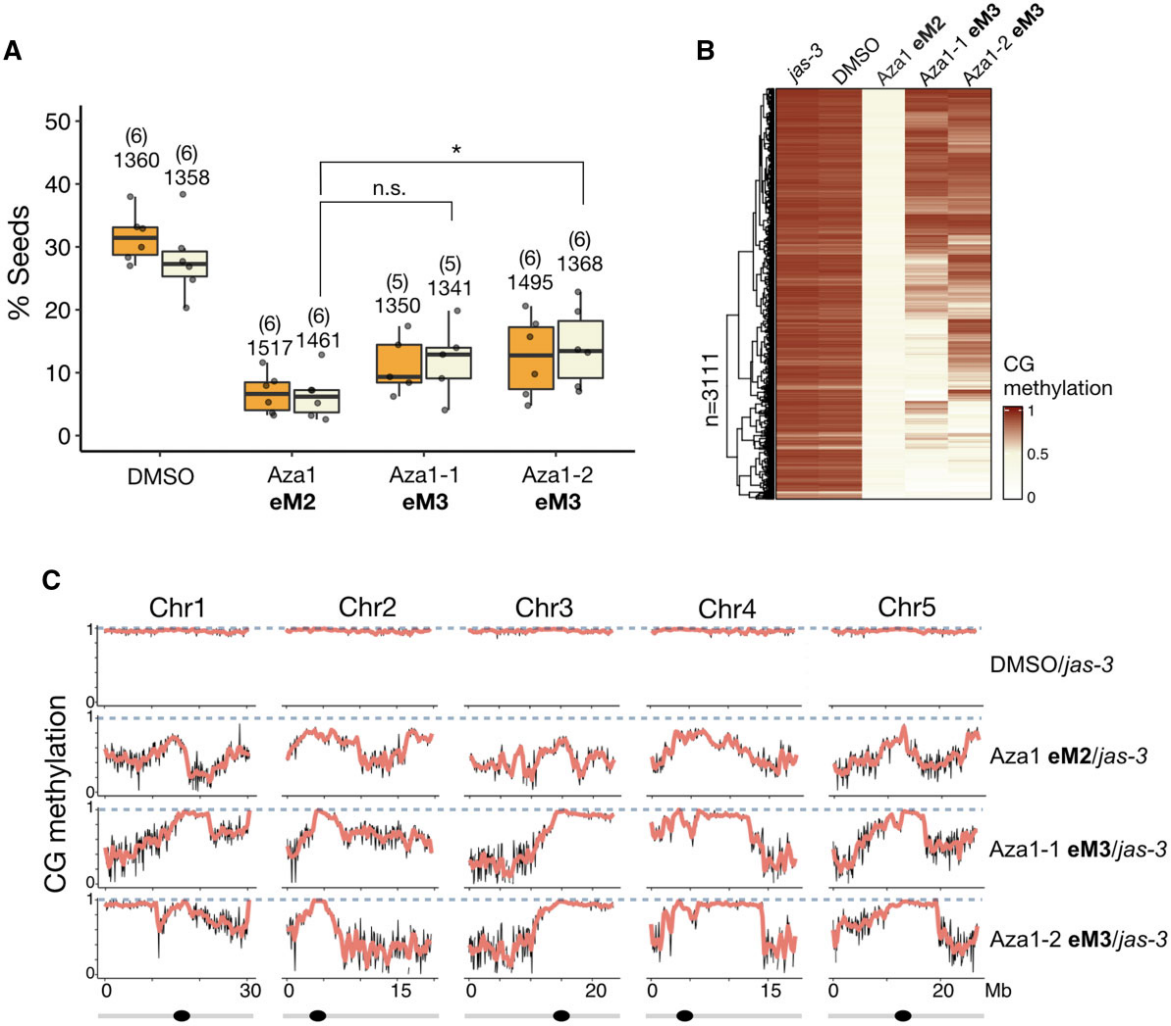
The triploid block response and CG methylation are partially restored two generations after treatment with 5-Azacytidine. A, Seed abortion (orange) and germination (ivory) assays were performed for two consecutive generations after treatment with 5-Azacytidine (eM2 and eM3), showing that the triploid block response is only partially restored in two independent lines in the third generation after epimutagenesis (eM3). Numbers above each box indicate the number of siblings (top) and total number of seeds (bottom) counted. Statistical significance was calculated by ANOVA with a post hoc Dunnett test, using Aza1 as the reference group (n.s. is not significant, *P ≤ 0.05). Boxes represent the interquartile range (IQR) showing the lower (Q1) and upper (Q3) quartiles surrounding the median (central line), and whiskers represent the minimum (Q1 −1.5*IQR) and maximum (Q3 + 1.5*IQR) values. B, Average CG methylation mapping to hypomethylated CG DMRs detected between the suppressor line Aza1 and the untreated control *jas-3* is presented as a heatmap for untreated jas-3 (two replicates) and DMSO (two replicates), Aza1 eM2, and two eM3 lines (Aza1-1 and Aza1-2), showing that CG methylation is partially restored in eM3 lines at these regions. C, CG methylation of untreated *jas-3*, DMSO, eM2, and eM3 lines was mapped to 100-kb bins across the Arabidopsis genome. CG methylation levels in the DMSO, eM2, and eM3 datasets were then divided by the levels in *jas-3* and plotted separately for all five chromosomes to show patterns of hypomethylation. This shows that for most of the genome, the distribution of CG hypomethylation is identical between the two eM3 lines, although there are particular loci where CG methylation is segregating. A schematic of each chromosome is illustrated below the metaplots, showing approximate centromere locations as black dots.

Epigenetic variation induced by 5-Azacytidine is known to be a transient effect (Pecinka and Liu, 2014), and only a few epialleles have been detected in subsequent generations after treatment (Akimoto et al., 2007). However, we found approximately 3,000 hypomethylated CG DMRs in the eM2 generation of strong suppressor lines (Figure  2C; Supplemental Figure S6 and Supplemental Data Set S2), which confirms that hypomethylation induced by 5-Azacytidine is stable for at least one generation after treatment. To further investigate the transgenerational stability of the suppressive effect, two diploid eM2 siblings from the strongest suppressor Aza1 were selected and selfed (Aza1-1 and Aza1-2), and six diploid eM3 plants were analyzed individually for each line (Supplemental Figure S3). Analysis of seed abortion and germination showed clear signs of recovery of the triploid block response in both lines, although only Aza1-2 showed significantly higher levels of nongerminated seeds than the levels detected in the eM2 generation (Figure  3A). We also performed WGBS analysis of bulked seedlings from each eM3 line. CG methylation was restored at approximately 35% of loci in these lines (Figure  3B), while ectopic CHG methylation was no longer detected (Supplemental Figure S7). These results strongly suggest that a progressive recovery of CG methylation contributes to the stronger triploid block response in eM3 plants. However, additional analyses will be required to determine if DNA methylation and the triploid block response are fully restored to *jas-3* levels in subsequent generations.

Interestingly, some DMRs segregated in the eM3 generation, thus suggesting allelic variations in DNA methylation levels in the progenitor line Aza1 eM2. This includes a large region in chromosome 1 whose CG methylation level was restored almost completely in Aza1-2, but remained hypomethylated in Aza1-1 (Figure  3C). Perhaps such differences explain the higher levels of triploid block in Aza1-2 (Figure  3A), although in the majority of DMRs (67% in Aza1-1 and 56% in Aza1-2), at least 80% of CG methylation that was lost in the progenitor line Aza1 eM2 was restored (Figure  3, B and C), thus indicating that certain epigenetic states were already fixed. Further analyses of the methylomes of additional lines and in different generations will be required for a robust evaluation of epiallele segregation in this population. However, since the triploid block response remained relatively low in eM3 lines compared to the DMSO controls (Figure  3A), we can conclude that DMRs with fully restored CG methylation in eM3 are likely not major contributors to the suppressive effect observed in the eM2 generation.

### Paternally expressed genes are transiently downregulated in the suppressor lines

**Figure 4:**
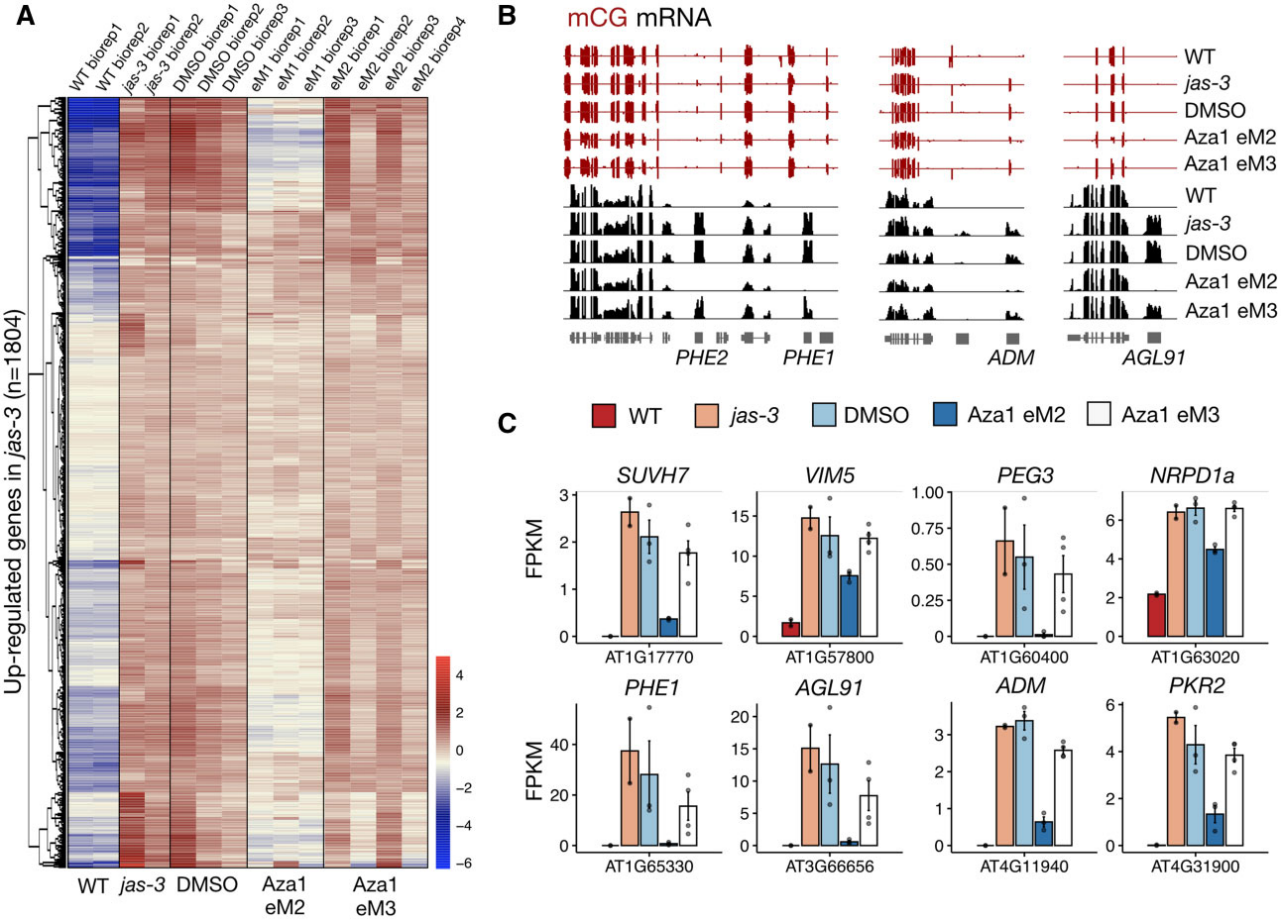
Paternally expressed genes are downregulated in the suppressor lines. A, Differential expression analysis was performed between WT Col-0 and *jas-3* mutant siliques, and 1,804 genes were found to be upregulated in *jas-3* (fold change ≥2, adjusted *P* <0.01). Transformed raw counts for these genes are presented as a heatmap for WT, *jas-3* and DMSO, Aza1 eM2, and eM3 biological replicates (see “Materials and Methods”), to show that a proportion of genes that are upregulated in the untreated *jas-3* and DMSO controls are transiently downregulated in the first generation after treatment with 5-Azacytidine. B, Genome browser tracks display CG methylation (pooled seedlings) and mRNA levels (siliques) in WT, *jas-3*, DMSO, Aza1 eM2, and Aza1 eM3 plants. C, Expression of selected PEGs was normalized as fragments per kilobase per million and plotted as barplots showing individual values (dots), mean, and error bar (*n*=2 for WT and *jas-3*, *n*=3 for DMSO and Aza1 eM2, *n*=4 for Aza1 eM3).

Previous studies in Arabidopsis and crops have shown that the triploid block leads to striking changes in the gene expression program of the developing triploid seed (Stoute et al., 2012; Schatlowski et al., 2014). Most notably, the expression of many PEGs is upregulated in abortive triploid seeds and is restored to wild-type (WT) diploid levels when the triploid block is genetically or epigenetically suppressed (Kradolfer et al., 2013; Schatlowski et al., 2014; Wolff et al., 2015; Martinez et al., 2018; Batista et al., 2019; Satyaki and Gehring, 2019). Therefore, we performed transcriptome analysis by mRNA sequencing of developing siliques collected 6–9 days post anthesis, as previously described (Mizzotti et al., 2018). Comparisons between WT Col-0 and *jas-3* mutant siliques revealed 668 downregulated and 1,804 upregulated genes in *jas-3* (Figure  4A; Supplemental Data Set S3). Similar analysis between WT and DMSO control plants showed a strong overlap between upregulated genes in untreated *jas-3* and DMSO control plants (Supplemental Figure S8 and Supplemental Data Set S3). These observations, together with the strong correlation between *jas-3* and DMSO replicates (Supplemental Figure S9), show that DMSO treatment does not induce major changes in the transcriptome of developing *jas-3* siliques.

We then compared suppressor Aza1 plants from the eM2 generation with the DMSO controls, finding that 470 genes were downregulated in Aza1 eM2, while only 48 were upregulated (fold change ≥2, *P *<* *0.01) (Supplemental Data Set S3). As expected, the vast majority of downregulated genes in Aza1 eM2 plants (93%) overlapped with upregulated genes in *jas-3* (versus WT; Supplemental Figure S8). We also performed transcriptome profiling in siliques of four different Aza1 siblings in the eM3 generation, as CG methylation and the triploid block response were partially restored in these plants (Figure  3). Indeed, 368 genes were significantly upregulated in eM3 siliques compared to eM2, while only 28 genes were downregulated in eM3 (fold change ≥2, *P *<* *0.01). Strikingly, most of the 368 upregulated genes in eM3 (90%) overlapped with the downregulated genes in eM2 plants (versus DMSO; Supplemental Figure S8). Indeed, PEGs are significantly represented in this list (Supplemental Figure S8; Pignatta et al., 2014; Wolff et al., 2011; Gehring et al., 2011; Hsieh et al., 2011; Del Toro-De León and Köhler, 2019), including the well-known genes *PHERES1* (*PHE1*) and *ADMETOS* (*ADM*), which have been associated with the triploid block (Figure  4, B and C; Kradolfer et al., 2013; Batista et al., 2019), whose expression was partially restored in the eM3 lines (Figure  4, B and C). We then compared these results with the lists of DMRs detected in the suppressor line Aza1 eM2 and found that 42 genes were overlapping or within 1-kb proximity (Supplemental Figure S10 and Supplemental Table S1). These genes are potential candidates to explain triploid block suppression in plants treated with 5-Azacytidine.

### Chemically induced hypomethylation allows interploidy and interspecific hybridization barriers to be bypassed

**Figure 5:**
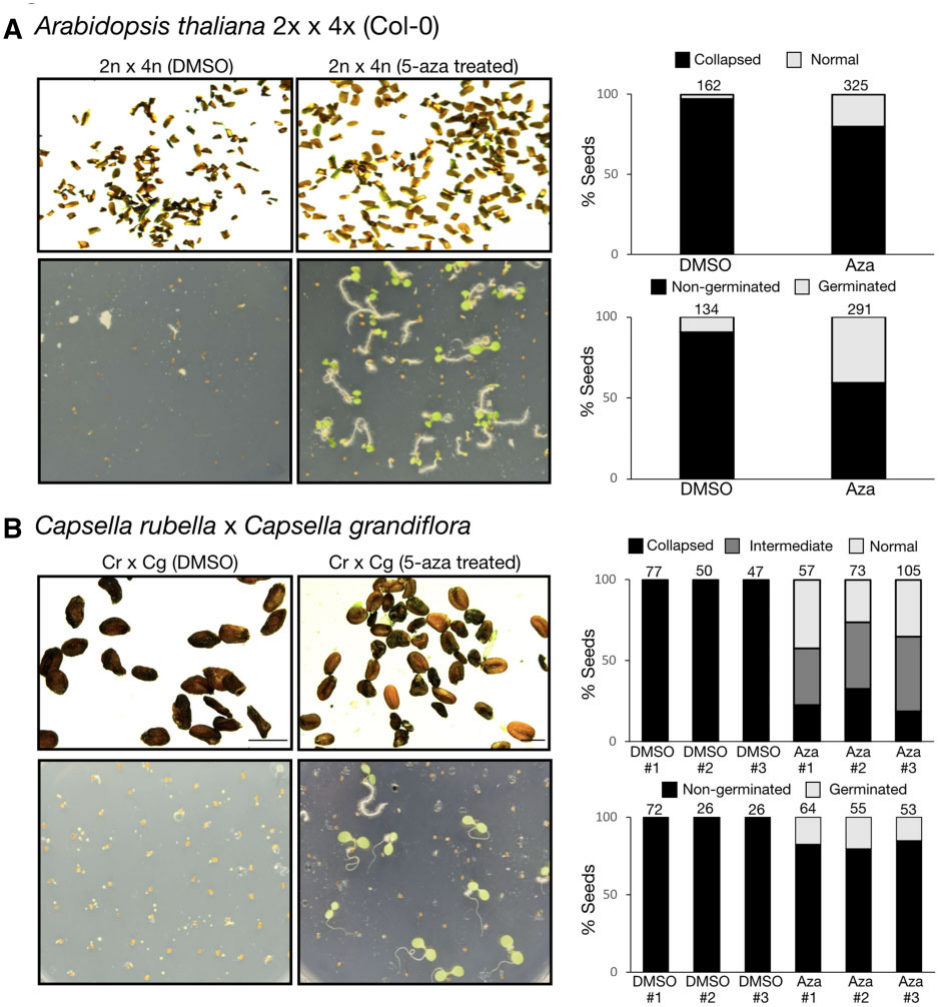
Chemically induced epimutagenesis allows interploidy and interspecific hybridization barriers to be bypassed. A, Pollen from tetraploid Arabidopsis plants in the Col-0 background was used to pollinate diploid Col-0 plants. Triploid seeds resulting from this cross aborted at high frequencies when the paternal parent was treated with DMSO, while 40% of viable triploid seeds were detected when pollen was derived from plants that had been treated with 5-Azacytidine. Barplots show the percentage of collapsed seeds (upper) and germination (lower) for each cross. B, In interspecific crosses between diploid *Capsella rubella* (*Cr*) and *Capsella grandiflora* (*Cg*), hybrid seeds aborted, resembling interploidy hybridizations with paternal excess. When *C. grandiflora* plants were treated with 5-Azacytidine, ∼20% of hybrid seeds germinated. Barplots show the percentage of collapsed seeds (upper) and germination (lower) for each cross. Scales bars represent 750 µm.

Endosperm-based hybridization barriers are frequently observed during interploidy and interspecific crosses in a variety of plant species (Lafon-Placette and Köhler, 2016). We therefore asked if genome-wide epimutagenesis induced by 5-Azacytidine could suppress hybridization barriers in crosses between plants of different ploidy or between different species. To test this hypothesis, we first treated tetraploid Arabidopsis plants in the Col-0 background with 100-µM 5-Azacytidine during seed germination. In agreement with the results in *jas-3* mutants, we obtained 40% viable triploid seeds when using pollen from 5-Azacytidine-treated tetraploid plants to pollinate nontreated diploid Col-0 plants (Figure  5A), while only 10% of triploid seeds were viable in the control cross using pollen from DMSO-treated plants. These results show that triploid block suppression after treatment with 5-Azacytidine is not dependent on the method used to produce diploid pollen.

We then performed interspecific hybridizations between *Capsella rubella* and *Capsella grandiflora*, as crosses between these species resemble the triploid block when *C. grandiflora* is used as the male parent (Rebernig et al., 2015). *Capsella* *grandiflora* plants were treated with 100-µM 5-Azacytidine and pollen from treated plants was used to pollinate nontreated *C. rubella* plants. Strikingly, ∼30% of hybrid seeds were plump and appeared normal, and ∼40% of seeds were less severely collapsed compared to the control DMSO crosses, which resulted in 100% completely collapsed seeds (Figure  5B). When these hybrid seeds were tested in germination assays, ∼15%–20% of hybrid seeds could germinate in the crosses where the male parent was treated with 5-Azacytidine (Figure  5B), while none of the seeds germinated in the control cross. Collectively, our data show that DNA hypomethylation induced by 5-Azacytidine treatment can suppress hybrid seed lethality in both interploidy and interspecific crosses.

## Discussion

The triploid block is a classic example of dosage regulation. Our recent studies have demonstrated how plants use epigenetic mechanisms to sense and control parental genome dosage in crosses between parents with differing ploidy (Schatlowski et al., 2014; Jiang et al., 2017; Borges et al., 2018; Martinez et al., 2018; Wang et al., 2018; Satyaki and Gehring, 2019; Dziasek et al., 2021; Florez-Rueda et al., 2021). Differential DNA methylation between parental genomes is essential for seed development and genomic imprinting (Adams et al., 2000; Choi et al., 2002; Xiao et al., 2006), and DNA methylation at the paternal genome is required to trigger the triploid block in Arabidopsis (Schatlowski et al., 2014; Satyaki and Gehring, 2019; Wang et al., 2021). However, and despite the recent advances, the mechanistic aspects of this complex process remain largely unclear.

In this study, we demonstrate that the triploid block is bypassed in epimutagenized plants treated with the cytosine analog 5-Azacytidine, which induces DNA hypomethylation. We found that the suppression level was highly correlated with a genome-wide and transgenerational loss of DNA methylation that occurred mostly in the CG context (Figure  2; Supplemental Figure S4). Interestingly, independent suppressor lines showed a strong loss of CG methylation at different loci, while nonsuppressors had almost normal levels of CG methylation (Figure  4B). The reason for this variability among treated plants remains to be explored, but one possibility is that only a fraction of plants treated with 5-Azacytidine are able to transmit DNA hypomethylation to their germline, and from there to the next generation.

We were able to validate previous results showing that DNA hypomethylation of the paternal genome is what allows triploid seeds to be viable (Schatlowski et al., 2014; Satyaki and Gehring, 2019; Wang et al., 2021). In previous studies, ectopic CHG methylation at PEGs was observed in the endosperm carrying a hypomethylated paternal genome derived from diploid *met1* pollen and was associated with triploid block suppression (Schatlowski et al., 2014). However, only one of the suppressor lines in our study showed a small number of loci with ectopic CHG methylation (Supplemental Figure S6), thus suggesting that this mechanism is unlikely responsible for PEG suppression in 5-Azacytidine-treated plants. Nevertheless, DNA methylation analyses in the developing endosperm of different suppressor lines will be required to fully evaluate this hypothesis.

Notably, we found a significant number of DMRs overlapping between the three strongest suppressors lines Aza1, Aza18, and Aza25 (Figure  2D). It is tempting to speculate that a particular epiallele (or epialleles) involved in the triploid block is within this list of 253 DMRs (Supplemental Data Set S2), although there is no clear overlap with genes previously associated with the triploid block (Supplemental Data Set S2). However, among all CG and CHG DMRs detected in the strong suppressor line Aza1 eM2, we identified 42 differentially expressed genes that could explain the suppressive effect (Supplemental Table S1 and Supplemental Figure S10). Alternatively, triploid block suppression in epimutagenized plants may simply require that a certain amount of the genome remains hypomethylated, independent of location. Comparisons between plants in the eM2 and eM3 generations support this idea, as DNA methylation was restored at only a fraction of hypomethylated CG DMRs detected in eM2 (Figure  3B), but the majority of genes that were downregulated in eM2 were significantly upregulated in eM3 (Supplemental Figure S7). Thus, genome-wide CG methylation levels of the paternal genome might somehow function as a “ploidy sensor” in the developing endosperm by attracting or repulsing epigenetic modulators of genomic imprinting. The most obvious candidates for this interplay are the paternal DNA methylation and maternal PRC2 pathways, which seem to be mutually exclusive at certain loci (Deleris et al., 2012; Zervudacki et al., 2018; Rougée et al., 2020) and have been independently implicated in the triploid block (Erilova et al., 2009; Martinez et al., 2018; Satyaki and Gehring, 2019; Wang et al., 2021).

Our work also shows that chemically induced epimutagenesis allows interspecific hybridization barriers to be bypassed in crosses between the Capsella species *C. rubella* and *C. grandiflora* (Figure  5B). Interestingly, CHG and CHH methylation was shown to decrease in Capsella hybrid endosperm, while CG methylation increased (Dziasek et al., 2021). However, it remains to be explored whether the increase in CG methylation is what causes hybrid seed defects in interspecific crosses, rather than the decrease in CHG and CHH methylation.

In conclusion, our study demonstrates that 5-Azacytidine can be successfully used as a tool to facilitate the generation of triploid seeds and interspecific F1 hybrids in different plant systems. We believe this method could be applicable to a wide range of species, including crops of high agronomic interest, thus providing a convenient and cheap strategy to facilitate modern plant breeding.

## Materials and methods

### Plant growth and chemically induced epimutagenesis

The mutant *A.* *thaliana* allele *jas-3* (SAIL_813_H03, Col-0 background) was used in this study. Diploid seeds from *jas-3* mutants were surface sterilized with 50% bleach for 5 min, rinsed with sterile deionized water, sown on agar plates containing 0.5X Murashige and Skoog (MS) medium, 1% sucrose, pH = 5.7, and different concentrations (25, 50, and 100 µg·mL^−1^) of 5-Azacytidine (Sigma), and placed in a growth chamber at 23°C, 70% humidity, 120 μE m^−2^ light with a 16-h light/8-h dark (long days) photoperiod. DMSO solvent was used as a control treatment. Seedlings were transferred to soil after 2 weeks and maintained in a greenhouse under long-day conditions to complete the lifecycle.

### Triploid block quantification

Dry seeds from five siliques were collected and imaged under a stereoscopic microscope (Nikon), and the triploid block was quantified by counting the number of aborted seeds. The same set of seeds was then surface-sterilized using 50% bleach and ethanol, rinsed once with ethanol 96%, and air-dried. The seeds were sown on agar plates containing 0.5X MS medium, 1% sucrose, pH = 5.7, stratified for 2 days at 4°C, and transferred to growth chambers at 23°C, 70% humidity, 120 μE m^−2^ light with a 16-h light/8-h dark (long days) photoperiod germination rate was initially quantified on 4- to 5-days-old seedlings, then adjusted after 7 days if necessary to account for germination delays.

### Analysis of pollen ploidy by flow cytometry

Pollen ploidy in the *jas-3* mutants was analyzed by collecting open flowers from individual plants into Eppendorf tubes, vortexing in 2 mL of 100-mM sodium phosphate buffer (pH 7) for 3 min, and filtering through a 50-µm nylon mesh. Pollen populations are characterized by an elevated high angle scatter (SSC) and autofluorescence, which allows haploid (1*n*) and diploid (2*n*) pollen to be discriminated, as previously described (Erilova et al., 2009; Storme and Geelen, 2011). These two populations were gated and quantified (Supplemental Figure S2). For ploidy analysis of nuclei, leaf tissue was chopped in 2 mL of Galbraith buffer (45-mM MgCl_2_, 20-mM MOPS, 30-mM sodium citrate, 1% (v/v) Triton X-100, pH 7.0) using a razor blade, filtered through a 50-µm mesh, stained with SYBR Green dye (Lonza), and analyzed on a CyFlow Space flow cytometer (Sysmex).

### Whole-genome bisulfite sequencing and DNA methylation analysis

Bulked seeds from each Aza line, two *jas-3* and two DMSO plants were germinated on MS plates. Genomic DNA was isolated from ten pooled seedlings using a Quick DNA purification kit (Zymo), and library preparation and sequencing were performed by BGI Genomics (Hong Kong). Briefly, genomic DNA was fragmented by sonication, end-repaired, and ligated to methylated adaptors. After bisulfite treatment, bisulfite-treated fragments were PCR amplified and sequenced as paired-end 100-bp reads (PE100) with DNBSEQ technology (BGI). Pre-processed and high-quality reads were mapped to the TAIR10 genome using bismark with default settings for paired-end libraries (Krueger and Andrews, 2011), and all figures and downstream analysis were performed using R. DMRs in the CG and CHG contexts were defined as 100-bp bins containing at least four or five differentially methylated CGs or CHGs and with an absolute methylation difference of at least 0.4 or 0.35, respectively. Regions with ectopic CHG methylation were defined as 100-bp bins without methylated CHG in the control dataset (untreated *jas-3*), and containing at least three differentially methylated CHGs with an absolute methylation increase of at least 0.15 in the suppressor lines. Around 100-bp bins localizing within 200 bp of each other were merged, and bins that merged at least once were considered to be DMRs.

### RNA sequencing and analysis

Total RNA was extracted from three siliques 6–9 days after anthesis, as previously described (Mizzotti et al., 2018) using an RNeasy Plant Mini Kit (Qiagen) following the manufacturer’s recommendations for seed tissues (RLC buffer). Sequencing of messenger RNA was performed by BGI Genomics (Hong Kong) using DNBSEQ technology. High-quality raw reads were aligned to the TAIR10 genome using STAR (Dobin et al., 2013). Reads were counted and normalized using the R package DESeq2 (Love et al., 2014). We considered differentially expressed genes to be those displaying a log2 fold-change ≥2, and with an adjusted *P* < 0.01. Graphical outputs were produced using the R packages ggplot2, pheatmap, and ComplexHeatmap. The list of PEGs used in the comparative analysis is presented in Supplemental Data Set S4.

### Interploidy and interspecific hybridizations

Seeds of *C.* *rubella* (accession 48.21) and *C.* *grandiflora* (accession 23.5), as well as diploid and tetraploid Col-0 seeds were surface sterilized with 30% bleach and 70% ethanol, rinsed with distilled water, and sown on agar plates containing 0.5X MS medium and 1% sucrose. Seeds of *C. grandiflora* and tetraploid Col-0 were also sown on agar plates with 0.5X MS medium, 1% sucrose, and 100-µM 5-Azacytidine (Sigma). All plates were placed in a growth chamber with a long-day photoperiod (16 h and 22°C light, 8 h and 19°C darkness) with a light intensity of 110 µE. Seven-day-old seedlings were transferred to pots filled with sterile soil, and plants were grown in a growth chamber with 60% humidity and daily cycles of 16-h light at 21°C and 8-h darkness at 18°C with a light intensity of 150 µE. Flower buds were manually emasculated and pollinated after 2 days. Dry seeds were stored for 30 days for “after-ripening”. They were then surface sterilized and sown on agar plates containing 0.5X MS medium and 1% sucrose. Plates were stratified for 2 days at 4°C and then moved to the growth chamber. Germination rate was scored after 7 days in the growth chamber. The experiment was done in three biological replicates (each replicate contained the offspring of different parental plants).

### Statistical analysis

Statistically significant differences in the percentage of collapsed and nongerminated seeds (Figures  1, A and C and 3A; Supplemental Figure S4A) were calculated by one-way analysis of variance (ANOVA) with a post hoc Dunnett test, using the R packages “ggpubr” and “multcomp”. A Wilcoxon test was used to compare the mean values between the amount of diploid pollen (Supplemental Figure S1B), using the R package “ggpubr”. The statistical significance of the observed overlaps between differentially methylated 100-bp bins (SupplementalFigure S2D) and between differentially expressed genes (Supplemental Figure S8) was calculated using the R package “SuperExactTest” (Wang et al., 2015). These analyses are presented in Supplemental File S1.

### Accession numbers

Sequence data from this article can be found in the NCBI’s Gene Expression Omnibus under the following accession number: GSE179702. A summary of all bisulfite and RNA sequencing data generated in this study is presented in Supplemental Data Set S1.

## Supplemental data

The following materials are available in the online version of this article.

**Supplemental Figure S1.** Quantification of diploid pollen in *jas-3* plants.

**Supplemental Figure S2.** Ploidy analysis by flow cytometry.

**Supplemental Figure S3.** Schematic depicting *jas-3* epimutagenesis and transgenerational analysis of the triploid block in the suppressor lines.

**Supplemental Figure S4.** Suppression of the triploid block in *jas-3* plants treated with 5-Azacytidine is a paternal effect.

**Supplemental Figure S5.** CG, CHG, and CHH methylation profiles at protein-coding genes and transposable elements.

**Supplemental Figure S6.** Differentially methylated regions.

**Supplemental Figure S7.** Ectopic CHG methylation in the suppressor lines.

**Supplemental Figure S8.** Differentially expressed genes in the suppressor lines.

**Supplemental Figure S9.** Clustering of RNA-seq datasets.

**Supplemental Figure S10.** Differentially expressed genes overlapping or within 1-kb proximity of CG and CHG DMRs in Aza1 eM2.

**Supplemental Table S1.** Differentially expressed genes overlapping or within 1-kb proximity of CG/CHG DMRs in Aza1 eM2.

**Supplemental Data Set S1.** Summary of whole-genome bisulfite and RNA-seq datasets.

**Supplemental Data Set S2.** Lists of differentially methylated regions.

**Supplemental Data Set S3.** Lists of differentially expressed genes.

**Supplemental Data Set S4.** Paternally expressed imprinted genes identified in previous studies.

**Supplemental File S1.** Statistical analysis. 

## Supplementary Material

koab284_Supplementary_Data

## Notes

F.B. designed the study; J.H., K.D., K.P., and F.B. performed the experiments; J.H., T.W., and F.B. analyzed the data; C.K. provided materials and advised on experimental design; and F.B. wrote the manuscript with contributions from all the authors.

The author responsible for distribution of materials integral to the findings presented in this article in accordance with the policy described in the Instructions for Authors (https://academic.oup.com/plcell) is: Filipe Borges (filipe.borges@inrae.fr).
