# Supplementary material for: Bypassing reproductive barriers in hybrid seeds using chemically induced epimutagenesis
Source: Plant Cell. 2021 Nov 18;34(3):989–1001. doi: 10.1093/plcell/koab284 (PMC8894923; doi:10.1093/plcell/koab284)
Supplement: koab284_Supplementary_Data [file koab284_supplementary_data.zip › tpc.21.00631_SupplementaFile1.pdf]

Supplemental File S1. Statistical analysis

Sum Sq = Sum of squares; df = degrees of freedom; Mean Sq = Mean Squares

Figure 1A

	df	Sum Sq	Mean Sq	F-value	Pr(>F)
Treatment	4	2251	562.8	10.78	3.01e-07 ***
Residuals	95	4959	52.2		

Multiple Comparisons of Means: Dunnett Contrasts
Linear Hypotheses:

	Estimate	Std. Error	t value	Pr(>|t|)
DMSO - jas-3 == 0	-0.08833	2.79830	-0.032	1.0000
Aza25 - jas-3 == 0	-6.97444	2.52595	-2.761	0.0235 *
Aza50 - jas-3 == 0	-8.02762	2.68497	-2.990	0.0126 *
Aza100 - jas-3 == 0	-11.76943	2.18025	-5.398	<0.001 ***

Signif. codes: 0 '***' 0.001 '**' 0.01 '*' 0.05 '.' 0.1 ' ' 1

Figure 1C

	df	Sum Sq	Mean Sq	F-value	Pr(>F)
Treatment	4	2658.0	664.5	18.43	3.61e-07 ***
Residuals	25	901.1	36.0		

Multiple Comparisons of Means: Dunnett Contrasts
Linear Hypotheses:

	Estimate	Std. Error	t value	Pr(>|t|)
Aza1 - DMSO == 0	-21.596	3.466	-6.230	<0.001 ***
Aza5 - DMSO == 0	3.559	3.466	1.027	0.6961
Aza14 - DMSO == 0	-9.320	3.466	-2.689	0.0418 *
Aza26 - DMSO == 0	2.117	3.466	0.611	0.9287

Signif. codes: 0 '***' 0.001 '**' 0.01 '*' 0.05 '.' 0.1 ' ' 1

Figure 2D

Intersections	Degree	Observed Overlap	Expected Overlap	FE	P-value
jas-3_aza25	1	10405	NA	NA	NA
jas-3_aza18	1	16981	NA	NA	NA
jas-3_aza18 & jas-3_aza25	2	2784	148.2939	18.7735	0
jas-3_aza1	1	17495	NA	NA	NA
jas-3_aza1 & jas-3_aza25	2	3763	152.7826	24.6298	0
jas-3_aza1 & jas-3_aza18	2	4760	249.3419	19.0903	0
jas-3_aza1 & jas-3_aza18 & jas-3_aza25	3	1316	2.1775	604.3668	0

Figure 3A

	df	Sum Sq	Mean Sq	F-value	Pr(>F)
Treatment	3	3616	1205.5	47.85	1.32e-13 ***
Residuals	42	1058	25.2		

Multiple Comparisons of Means: Dunnett Contrasts

Linear Hypotheses:

	Estimate	Std. Error	t value	Pr(>|t|)
Aza1-1 - Aza1 == 0	5.599	3.262	1.717	0.1867
Aza1-2 - Aza1 == 0	7.675	3.110	2.468	0.0493 *

Signif. codes: 0 ‘***’ 0.001 ‘**’ 0.01 ‘*’ 0.05 ‘.’ 0.1 ‘ ’ 1

Supplemental Figure S1B

	statistic	p-value	p.signif	method
DMSO-Aza1	17	0.937	ns	Wilcoxon

Supplemental Figure S4A

	df	Sum Sq	Mean Sq	F-value	Pr(>F)
Treatment	3	2932.1	977.4	70.3	4.33e-06 ***
Residuals	8	111.2	13.9		

Multiple Comparisons of Means: Dunnett Contrasts

Linear Hypotheses:

	Estimate	Std. Error	t value	Pr(>|t|)
DMSOxAza1 - Aza1 == 0	-4.406	3.045	-1.447	0.388
Aza1xDMSO - Aza1 == 0	33.750	3.045	11.086	<0.001 ***

Supplemental Figure S8A

Intersections	Degree	Observed Overlap	Expected Overlap	FE	P-value
up in DMSO vs WT	1	1856	NA	NA	NA
up in jas-3 vs WT	1	1804	NA	NA	NA
up in jas-3 vs WT & up in DMSO vs WT	2	1584	123.0377	12.8741	0

Supplemental Figure S8B

Intersections	Degree	Observed Overlap	Expected Overlap	FE	P-value
up in Aza1 eM3 vs Aza1 eM2	1	368	NA	NA	NA
down in Aza1 eM2 vs DMSO	1	470	NA	NA	NA
down in Aza1 eM2 vs DMSO & up in Aza1 eM3 vs Aza1 eM2	2	331	6.3558	52.0785	0
up in jas-3 vs WT	1	1804	NA	NA	NA
up in jas-3 vs WT & up in Aza1 eM3 vs Aza1 eM2.	2	333	24.3954	13.6501	0
up in jas-3 vs WT & down in Aza1 vs DMSO.	2	436	31.1572	13.9936	0
up in jas-3 vs WT & down in Aza1 eM2 vs DMSO & up in Aza1 eM3 vs Aza1 eM2	3	323	0.4213	766.6077	0

Supplemental Figure S8C

Intersections	Degree	Observed Overlap	Expected Overlap	FE	P-value
PEGs	1	245	NA	NA	NA
down in Aza1 eM2 vs DMSO	1	470	NA	NA	NA
down in Aza1 eM2 vs DMSO & PEGs	2	38	4.2314	8.9804	5.122986e-25

]
