# Supplementary material for: Bypassing reproductive barriers in hybrid seeds using chemically induced epimutagenesis
Source: Plant Cell. 2021 Nov 18;34(3):989–1001. doi: 10.1093/plcell/koab284 (PMC8894923; doi:10.1093/plcell/koab284)
Supplement: koab284_Supplementary_Data [file koab284_supplementary_data.zip › tpc.21.00631_SupplementalFiguresandTables.pdf]

SUPPLEMENTAL DATA

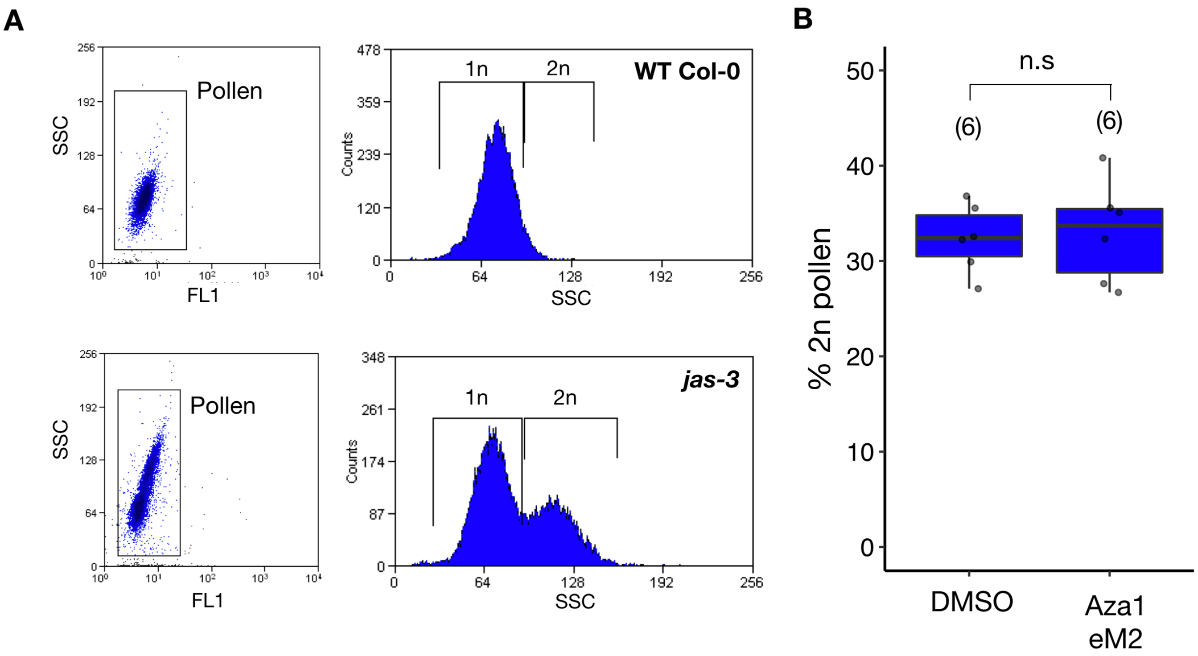

Supplemental Figure S1. Quantification of diploid pollen in *jas-3* plants. (Supports Figure 1)

(A) The proportion of haploid (1n) and diploid (2n) pollen in *jas-3* mutants was quantified by flow cytometry, as pollen populations are characterized by an elevated high angle scatter (SSC) and autofluorescence (FL1). Wild-type Col-0 plants do not show the peak corresponding to diploid pollen.

(B) Boxplot to show the distribution of diploid (2n) pollen among individual DMSO and Aza1 eM2 plants (*jas-3* background). Number above the boxes represent the number of plants used. A Wilcoxon test was used to compare the mean values between the suppressor Aza1 and the DMSO control. n.s. is not significant (p=0.937).

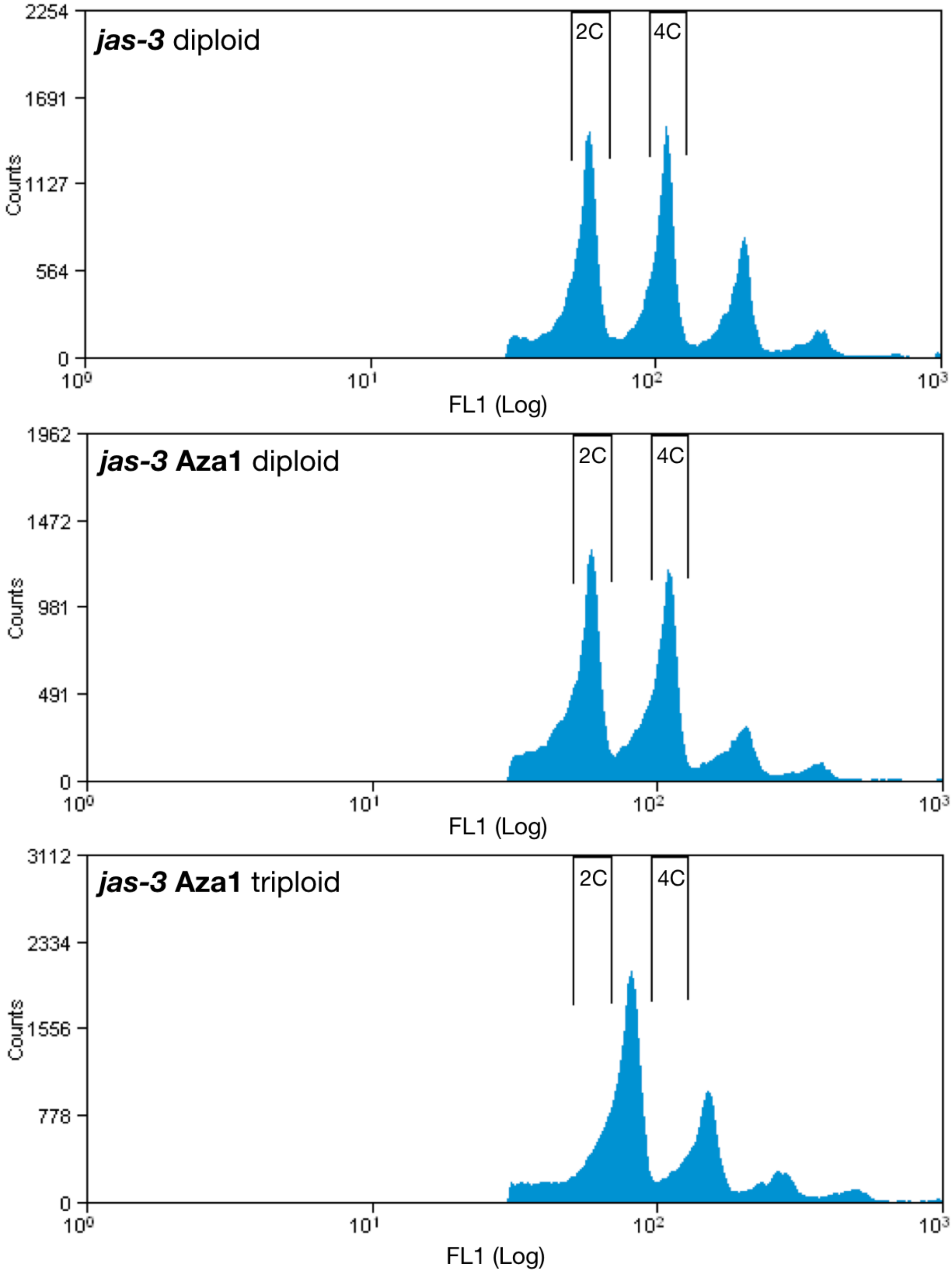

Supplemental Figure S2. Ploidy analysis by flow cytometry. (Supports Figure 1) Leaf tissue was chopped in Galbraith buffer with a razor blade, stained with SYBR Green dye (Lonza), and analyzed on a CyFlow Space cytometer (Sysmex). Nuclei of young diploid leaves in Arabidopsis is characterized by two prominent 2C and 4C peaks. Triploid individuals show a proportional increase in signal intensity.

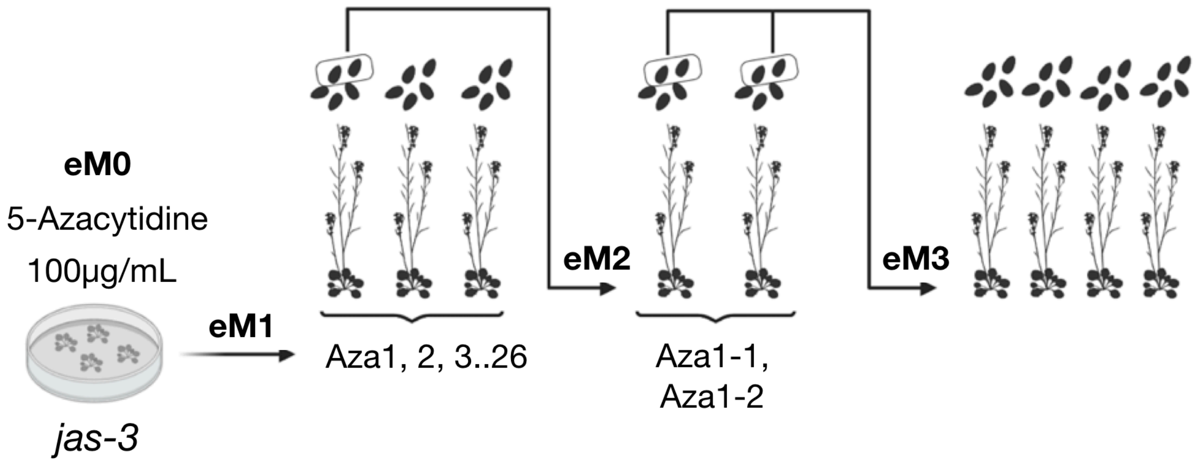

Supplemental Figure S3. Schematic depicting *jas-3* epimutagenesis and transgenerational analysis of the triploid block in the suppressor lines. (Supports Figures 1, 2, 3 and 4)

Diploid seeds from *jas-3* plants were treated with 100µg/mL of 5-Azacytdine during germination and early growth (eM0). Treated eM1 plants were then transferred to soil to recover and allowed to self-fertilize. Selfed *jas-3* mutants produce diploid seeds that are viable and triploid seeds that abort. Diploid plants were selected after each generation and allowed to self-fertilize, and the triploid block was quantified by counting the number of aborted seeds in individual siblings over two consecutive generation (eM2 and eM3).

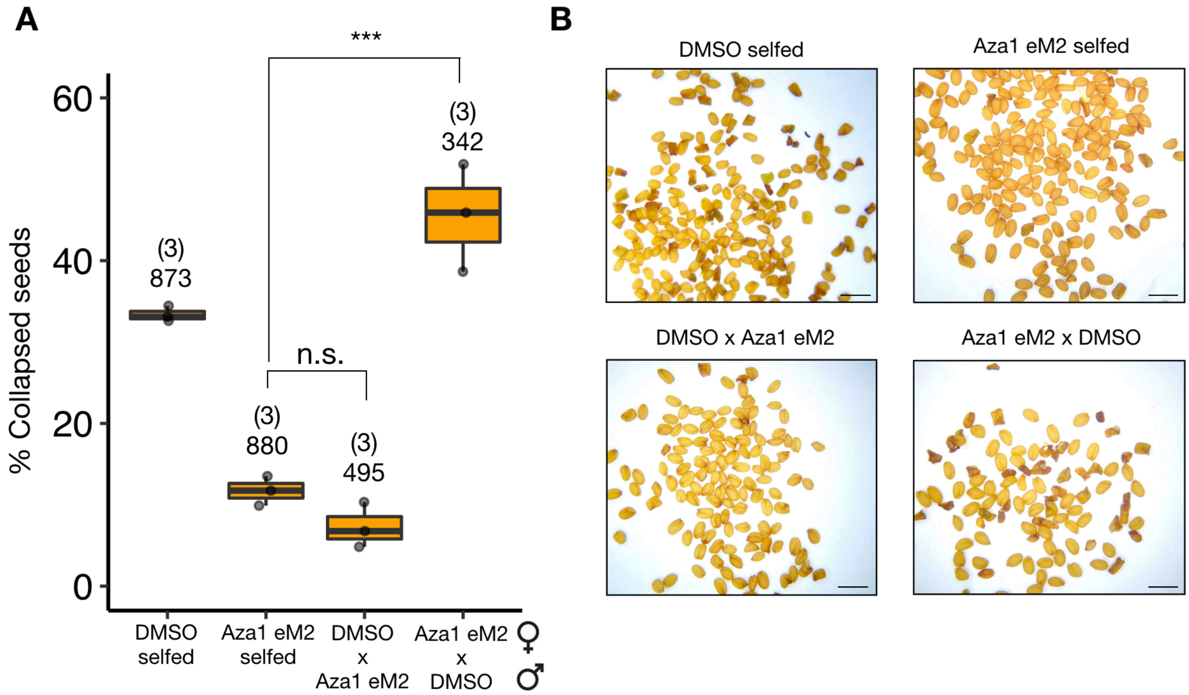

Supplemental Figure S4. Suppression of the triploid block in *jas-3* plants treated with 5-Azacytidine is a paternal effect. (Supports Figure 1)

(A) A parental effect in triploid block suppression after epimutagenesis was investigated by performing reciprocal crosses between siblings of the strong suppressor Aza1 in the eM2 generation and DMSO control plants. Numbers above each box represent the total number of plants used (top) and total number of seeds counted (bottom). Statistically significant differences in the percentage of collapsed seeds were calculated by ANOVA with a post hoc Dunnett test, using Aza1 eM2 as the reference group (n.s. is not significant, and *** is $p \leq 0.001$). Boxes represent the interquartile range (IQR) showing the lower (Q1) and upper (Q3) quartiles surrounding the median (central line), and whiskers represent the minimum (Q1 – 1.5*IQR) and maximum (Q3 + 1.5*IQR) values.

(B) Representative images of seeds are shown for the controls (selfed plants) and reciprocal crosses. Scale bars represent 1mm.

Supplemental Data. Huc et al. (2022). Bypassing reproductive barriers in hybrid seeds using chemically induced epimutagenesis. Plant Cell.

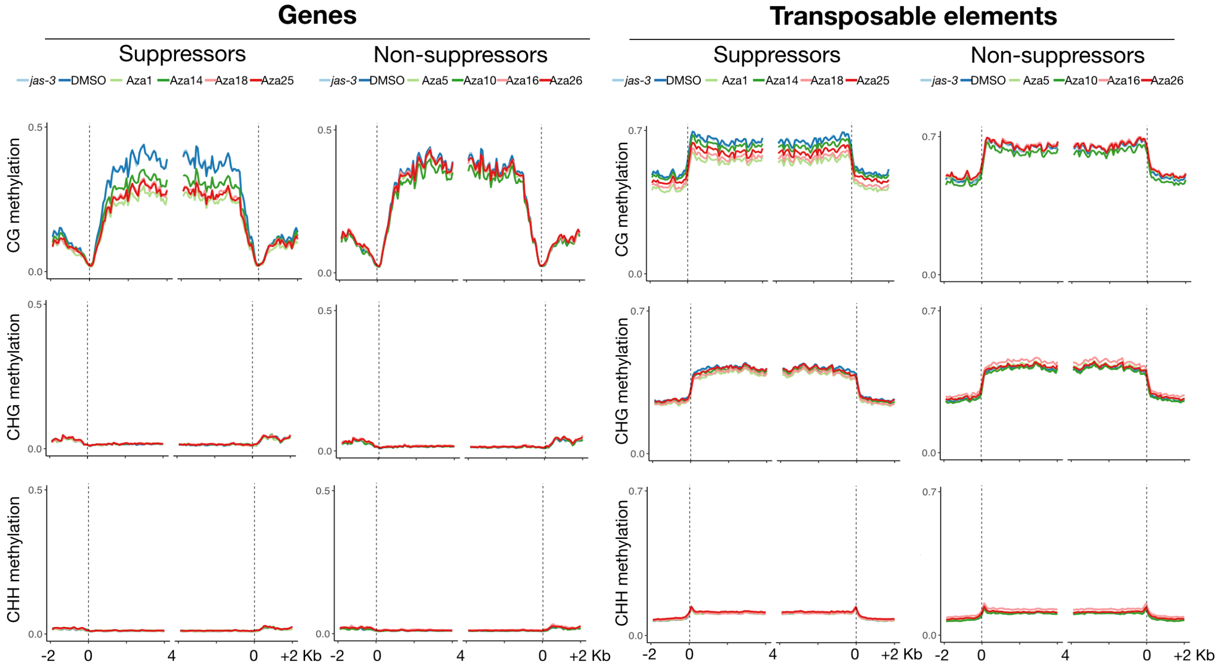

Supplemental Figure S5. CG, CHG, and CHH methylation profiles at protein-coding genes and transposable elements. (Supports Figure 2)

Protein-coding genes (left panels) and transposable elements (TEs, right panels) annotated according to the TAIR10 reference genome were aligned at the 5′ and 3′ ends (dashed lines), and average CG, CHG, and CHH methylation levels for 100-bp intervals were plotted for untreated *jas-3* and the DMSO controls and for suppressor and non-suppressor lines in the eM2 generation. The loss of DNA methylation at genes and TEs mainly occurred in the CG context and was observed only in the suppressor lines.

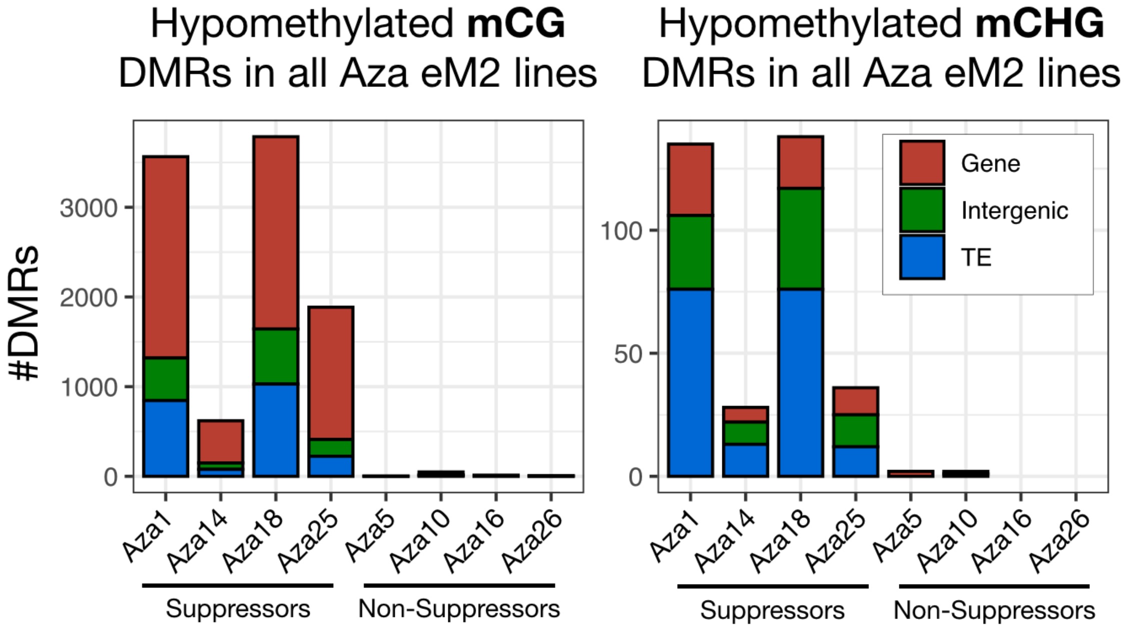

Supplemental Figure S6. Differentially methylated regions. (Supports Figure 2) Hypomethylated CG and CHG DMRs detected in the suppressor and non-suppressor lines in the eM2 generation were mapped to the genomic features annotated in the TAIR10 reference genome, showing that the majority CG and CHG DMRs overlap with protein-coding genes and transposable elements (TEs), respectively.

Supplemental Data. Huc et al. (2022). Bypassing reproductive barriers in hybrid seeds using chemically induced epimutagenesis. Plant Cell.

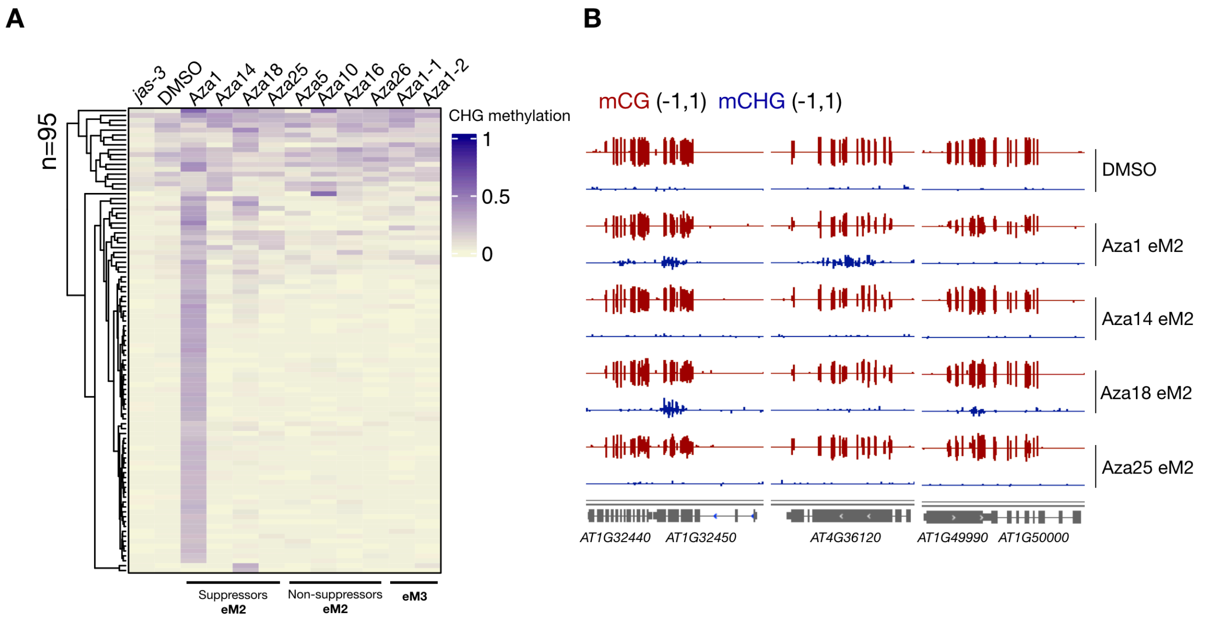

Supplemental Figure S7. Ectopic CHG methylation in the suppressor lines. (Supports Figure 2)

(A) Heatmap representation of CHG methylation levels at ectopic CHG DMRs detected in the suppressor line Aza1 eM2 compared to the untreated control *jas-3*. Average CHG methylation mapping to these DMRs is presented as a heatmap for two independent replicates of untreated *jas-3* and the DMSO controls, suppressor and non-suppressor lines in the eM2 generation, and the two eM3 lines.

(B) Genome browser tracks show DNA methylation in the CG and CHG contexts for selected loci showing ectopic CHG methylation in the suppressor lines compared to the DMSO control.

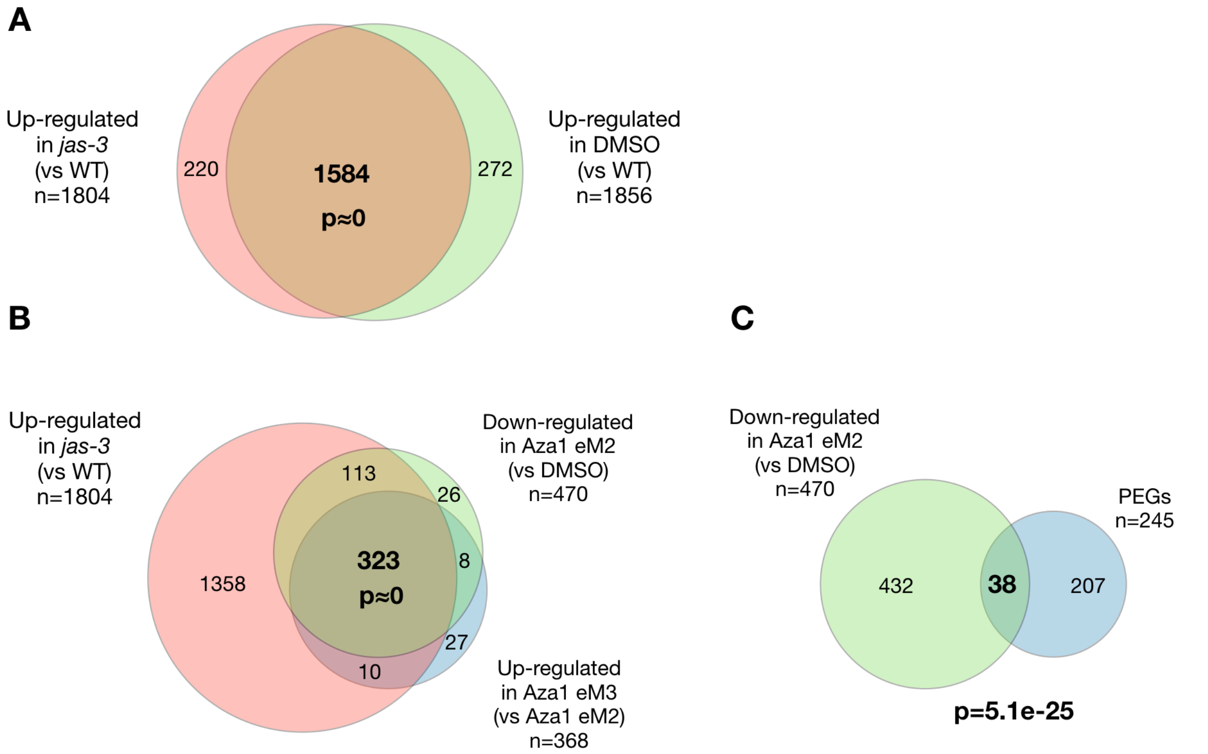

Supplemental Figure S8. Differentially expressed genes in the suppressor lines. (Supports Figure 4)

(A) Venn diagram shows the overlap between up-regulated genes in untreated *jas-3* (vs. WT), and up-regulated genes in the DMSO controls (vs. WT).

(B) Venn diagram shows the overlap of up-regulated genes in *jas-3* (vs. WT) with genes that were transiently down-regulated in eM2 (vs. DMSO) and up-regulated in eM3 (vs. eM2). The majority of genes that were down-regulated in eM2 were up-regulated in *jas-3* mutant siliques, and the expression of these genes was restored in eM3.

(C) Paternally expressed genes (PEGs) are significantly represented in the list of down-regulated genes in Aza1 eM2.

The statistical significance of the observed overlaps between differentially expressed genes was calculated using the R package SuperExactTest (Wang et al., 2015).

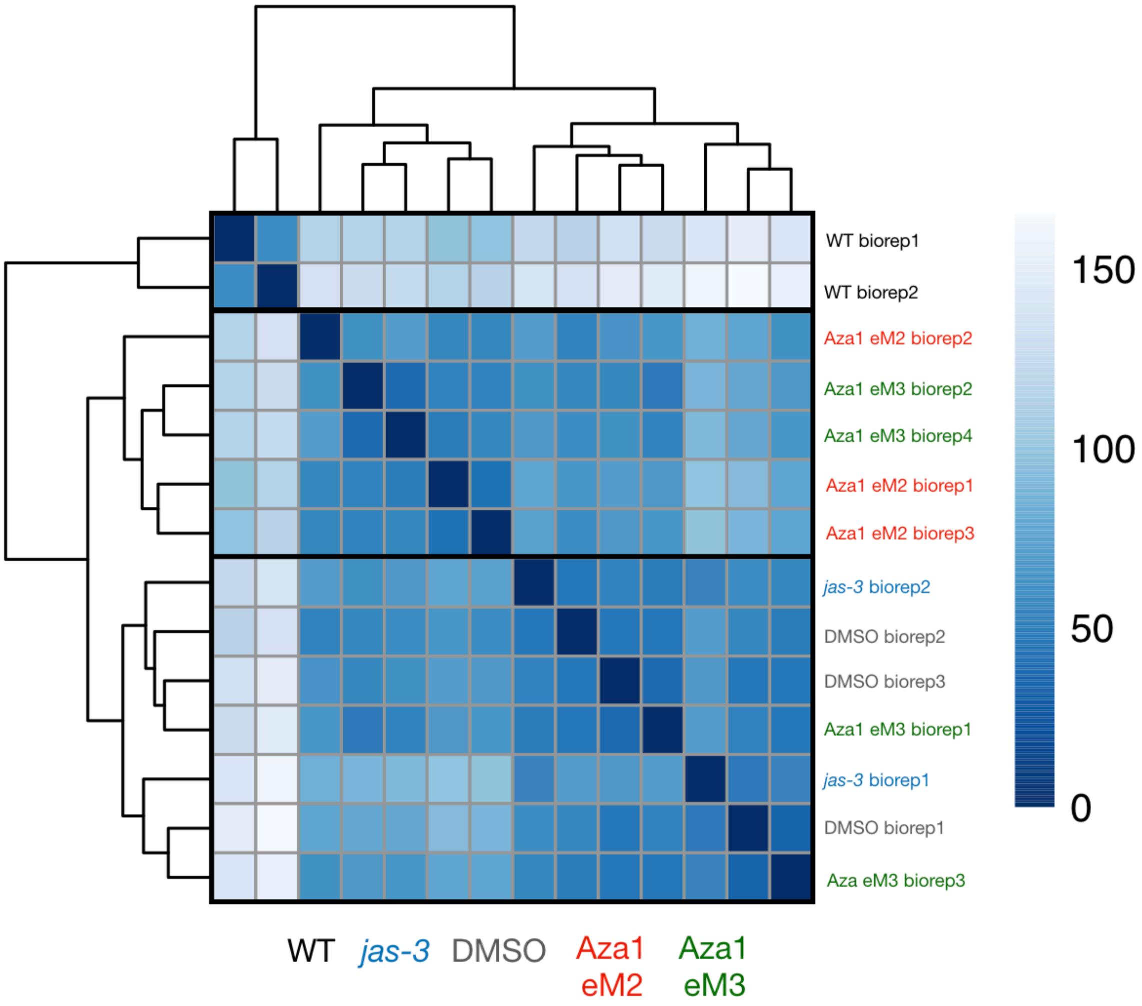

Supplemental Figure S9. Clustering of RNA-seq datasets. (Supports Figure 4) Transformed data were used for sample clustering to visualize sample-to-sample distances (see Methods). The scale gradient represents sample-to-sample distances. The heatmap of this distance matrix shows an overview of similarities and dissimilarities between all RNA-seq datasets produced in this study.

Supplemental Data. Huc et al. (2022). Bypassing reproductive barriers in hybrid seeds using chemically induced epimutagenesis**.** Plant Cell.

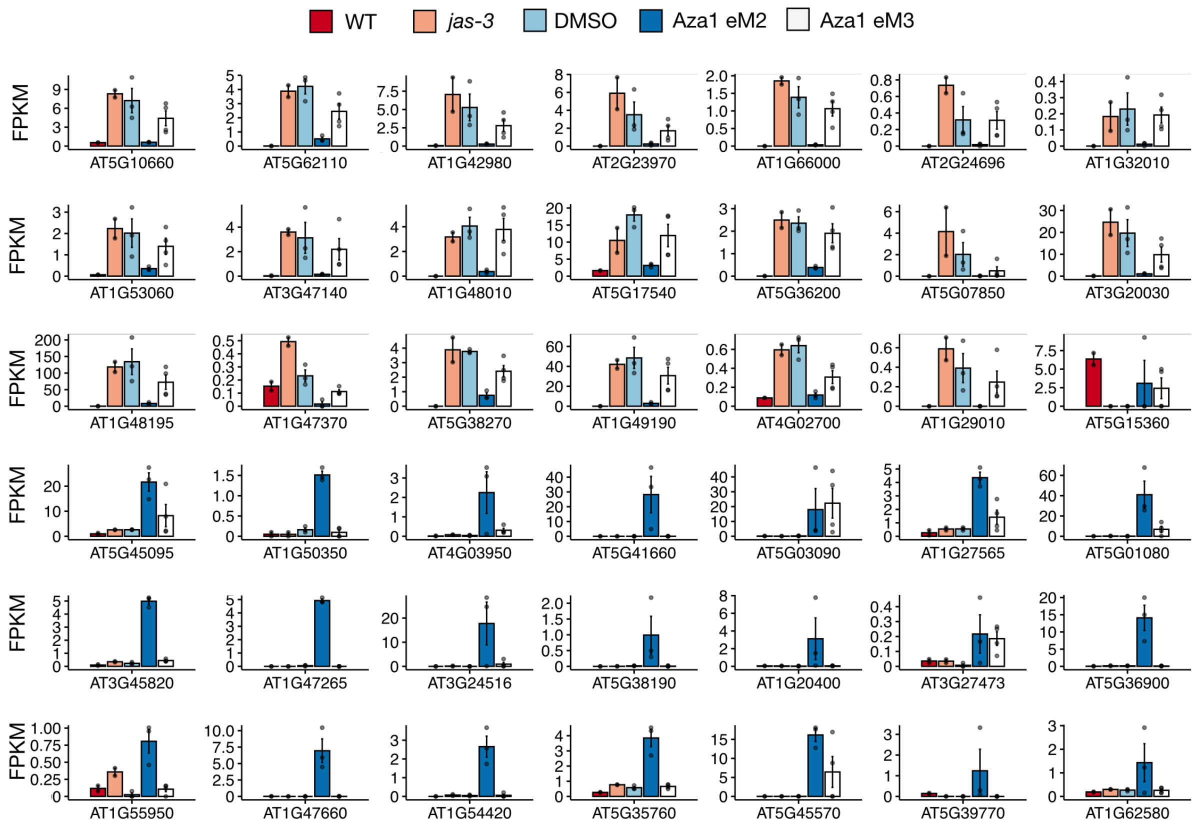

Supplemental Figure S10. Differentially expressed genes overlapping or within 1kb proximity of CG and CHG DMRs in Aza1 eM2.
Raw counts were normalized as fragments per kilobase per million (FPKM) and plotted as barplots showing individual values (dots), mean, and error bar (n=2 for WT and *jas-3*, n=3 for DMSO and Aza1 eM2, n=4 for Aza1 eM3).

Supplemental Table S1. Differentially expressed genes overlapping or within 1kb proximity of CG/CHG DMRs in Aza1 eM2.

Gene Identifier	Gene Model Description	Expression	DNA methylation
**AT5G10660**	calmodulin-binding protein-like protein	Down-regulated in Aza1 eM2	Ectopic CHG in Aza1 eM2
**AT5G62110**	Homeodomain-like superfamily protein		
**AT1G42980**	Actin-binding FH2 (formin homology 2) family protein		Hypo CG/CHG in Aza1 eM2
**AT2G23970**	Class I glutamine amidotransferase-like superfamily protein		
**AT1G66000**	hypothetical protein (DUF577)		
**AT2G24696**	transcriptional factor B3 family protein		
**AT1G32010**	myosin heavy chain-like protein		
**AT1G53060**	Legume lectin family protein		
**AT3G47140**	F-box associated ubiquitination effector family protein		
**AT1G48010**	Plant invertase/pectin methylesterase inhibitor superfamily protein		
**AT5G17540**	HXXXD-type acyl-transferase family protein		
**AT5G36200**	F-box and associated interaction domains-containing protein		
**AT5G07850**	HXXXD-type acyl-transferase family protein		
**AT3G20030**	F-box and associated interaction domains-containing protein		
**AT1G48195**	Zinc finger C-x8-C-x5-C-x3-H type family protein		
**AT1G47370**	RBA1 variant		
**AT5G38270**	F-box family protein		
**AT1G49190**	member of Response Regulator: B-Type		
**AT4G02700**	sulfate transporter 3		
**AT1G29010**	verprolin		
**AT5G15360**	transmembrane protein	Up-regulated in Aza1 eM2	
**AT5G45095**	hypothetical protein		
**AT1G50350**	E3 ubiquitin-protein ligase RING1-like protein		
**AT4G03950**	Nucleotide/sugar transporter family protein		
**AT5G41660**	transmembrane protein		

**AT5G03090**	Mto 1 responding down protein		
**AT1G27565**	hypothetical protein		
**AT5G01080**	Beta-galactosidase related protein		
**AT3G45820**	hypothetical protein		
**AT1G47265**	hypothetical protein		
**AT3G24516**	hypothetical protein		
**AT5G38190**	myosin heavy chain-like protein		
**AT1G20400**	hypothetical protein (DUF1204)		
**AT3G27473**	Cysteine/Histidine-rich C1 domain family protein		
**AT5G36900**	hypothetical protein		
**AT1G55950**	DNA-binding storekeeper protein-related transcriptional regulator		
**AT1G47660**	hypothetical protein		
**AT1G54420**	hypothetical protein		
**AT5G35760**	Beta-galactosidase related protein		
**AT5G45570**	Ulp1 protease family protein		
**AT5G39770**	Represents a non-function pseudogene homologous to AtMSU81 (At4g30870).		
**AT1G62580**	Encodes a flavin monooxygenase that binds NO		
